# German Dentists’ Preferences for the Treatment of Apical Periodontitis: A Cross-Sectional Survey

**DOI:** 10.3390/ijerph17207447

**Published:** 2020-10-13

**Authors:** Jonas Conrad, Jan Retelsdorf, Sameh Attia, Christof Dörfer, Mohamed Mekhemar

**Affiliations:** 1 Clinic for Conservative Dentistry and Periodontology, School of Dental Medicine, Christian-Albrecht’s University, 24105 Kiel, Germany; conrad@konspar.uni-kiel.de (J.C.); doerfer@konspar.uni-kiel.de (C.D.); 2 Department of General, Faculty of Education, Intercultural and International Comparative Education University of Hamburg, 20146 Hamburg, Germany; jan.retelsdorf@uni-hamburg.de; 3 Department of Cranio Maxillofacial Surgery, Justus-Liebig University, 35392 Giessen, Germany; sameh.attia@dentist.med.uni-giessen.de

**Keywords:** Root canal treatment, endodontic treatment, apical periodontitis, oral disease, health-related quality of life, German dentists, treatment decision

## Abstract

Currently, there is no standard treatment protocol for apical periodontitis (AP). Thus, restorable teeth might get extracted and replaced prosthetically. This study evaluated German dentists’ preferred AP treatment decisions and the influencing factors for selecting tooth retention by initial/repeated surgical/non-surgical root-canal treatment (RCT) or extraction with/without prosthetic replacement. Through an online-survey, participants (*n* = 260) rated different treatment options for four case scenarios with AP in anterior/posterior teeth without/with previous RCT. Statistical analysis included the Friedman test for intra-case comparisons and Chi-squared test for factor-associations (*p* ≤ 0.05). Tooth retention using initial/repeated RCT was ranked first in all scenarios and rated as (very) appropriate by most participants, while implant-supported crowns (ISC) and apicoectomy had the second ratings. ISC were preferred more on posterior teeth or previous root-canal-treated teeth. Rating levels of treatment options displayed significant differences for all case scenarios. Posterior tooth retention by RCT demonstrated a significant association with work experience. Tooth retention with previous RCT displayed a significant correlation with dentists’ privately insured patients. Most dentists preferred tooth preserving with initial/repeated RCT, while others selected non-evidence-based choices. This reflects a lack of consensus of AP treatment decisions in Germany. Fixed treatment guidelines and further evaluation of treatment–decision-correlated factors are recommended for correct treatment planning.

## 1. Introduction

Helping patients to keep a healthy and functional dentition is an ultimate aim of dentistry and public oral health. Besides being a marker of functional aging, patients’ loss of dentition has been linked with a higher occurrence of dementia, psychological distress, and a poor health related quality of life [1,2]. An important dilemma often discussed by clinicians is when teeth should be extracted and restored instead [3]. This problem should be increasingly recognized by the dental society as making treatment decisions that do not consider the patients’ preferences or quality of life can affect the public health severely.

Apical periodontitis (AP), a highly prevalent dental disease resulting from an endodontic infection of the root canal system, is one of the main reasons for tooth extraction [4]. The prevalence of AP increases during life and by the age of 50 years one in two individuals is affected by this disease [4]. Treatment options of AP include mainly root-canal treatment (RCT) or the extraction of the affected tooth [5]. In case of tooth extraction, the edentulous area can be restored by fixed or removable dental prostheses (FDP and RDP, respectively), implant supported crowns (ISC), or remains edentulous. Another possible treatment modality includes apicoectomy after an unsuccessful RCT [6].

Previous studies present different perspectives among dentists on the correct treatment plan for AP [4,7,8,9,10,11,12]. Besides several disagreements about radiographic analyses and radiographic based treatment decisions, dentists also display no agreement on treatment choices in different clinical circumstances. Furthermore, the investigations display that clinical decision-making may be reliant on the dentist’s clinical experience and education, as well as the working environment and oral health systems in the involved countries [4,7,8,13,14]. By identifying and addressing this gap among clinicians’ treatment decisions, an academic society can provide predictive guidelines and an effective development of skills and knowledge to the dentists (as required) to preserve and improve the oral public health and quality of life of the patients.

Earlier investigations have compared clinical decisions for the treatment of AP among dentists in different countries using survey models [4,7,8,9,10,12]. Several surveys involved investigations of patients’ periapical radiographs under a specified clinical scenario, after which the dentists chose the preferred treatment plan. Other surveys sent diverse clinical scenarios to different dentist groups and revealed that clinical backgrounds and experiences, as well as factors related to the region of dental education and practice can affect the chosen treatment plan for AP. To the best of our knowledge, there is no definite treatment protocol for teeth with AP, raising the concerns that some affected but restorable teeth might get extracted and affect the oral health-related quality of life of patients negatively [5,15,16].

In Germany, all residents are required by law to have a health insurance. Nearly 90% of the inhabitants have statutory health insurance and about 10% are privately insured. Statutory insurers basically offer the same standard medical treatments, prescription drugs, and health care services. The standard benefits likewise include dental check-ups, dental and gingival treatment, as well as orthodontic procedures. When it comes to dental implants and prosthetics, statutory health insurers recompense a fixed amount. Before starting any prosthetic or implant-associated dental work, the dentist provides a treatment and cost plan that must be submitted to the insurer. The insurer afterwards decides the costs covered and how much the patient has to pay by himself. Private health insurers may cover special services as treatment by a senior consultant or head of department, having a private hospital room, as well as special dental treatments including prostheses and dental implants. The included private insurance benefits are specified in the individual policy [17].

Thus, the aim of the current survey was to evaluate German dentists’ preferences for the treatment of teeth with AP in northern Germany via four different case scenarios including the selection of tooth retention using RCT compared to tooth extraction followed by replacement with ISC/FDP/RDP or no replacement. Furthermore, this survey intended to investigate whether various influencing factors are associated to the first-ranked treatment option selected for each of the different case scenarios presented in the survey.

## 2. Materials and Methods 

### 2.1. Study Population and Methodology 

In the current study, data on dentists’ treatment selection preferences was collected using an online survey as previously described [10] with slight modifications in the available clinical scenarios to adapt to the German oral healthcare system. The questionnaire was translated into the German language by an authorized translation expert and was pilot tested by a focus group of ten dentists from northern Germany. The focus group represented dentists from private practices and university clinics. It included variations of gender, age, as well as in work and educational experience. Furthermore, the survey was validated [18] by five experts in the fields of dentistry, psychology, and web-based surveys at the University of Kiel and transformed into an online survey using a web-based survey tool (Unipark, QuestBack GmbH, Cologne, Germany). After the approval by the University of Kiel Ethics Board (D499/16) the survey link was sent by email to all registered dentists at the state dental council (Zahnärztekammer Schleswig-Holstein, Kiel, Germany) in the northern state of Schleswig-Holstein, Germany (*N* = 2270). The introductory text in the email briefly explained the research project and assured anonymity and voluntary participation to the dentists. No financial incentives or gifts were promised to the participants and no exclusion criteria were defined (e.g., age, gender, or nationality).

In the first part of the questionnaire participants’ sociodemographic data such as gender, work experience, current employment situation, specialty education, location of their practice/workplace, performed treatment spectrum, and the percentage of privately insured patients in their dental practice/workplace was gathered. 

In the second part participants were provided with four written, clinical scenarios of a 50-year-old patient suffering from an AP on a permanent tooth:

Case Scenario 1 (CS1)—AP on a permanent, anterior tooth with no root canal filling;
Case Scenario 2 (CS2)—AP on a permanent, posterior tooth with no root canal filling;
Case Scenario 3 (CS3)—AP on a permanent, anterior tooth with root canal filling;
Case Scenario 4 (CS4)—AP on a permanent, posterior tooth with root canal filling.

For CS1 and CS2, participants were asked to assess the following treatment options according to their preferences:

1. Tooth retention using RCT followed by a coronal restoration;
2. Tooth retention using apicoectomy followed by a coronal restoration;
3. Tooth extraction followed by replacement with ISC;
4. Tooth extraction followed by replacement with FDP;
5. Tooth extraction followed by replacement with RDP;
6. Tooth extraction followed by no replacement.

For CS3 and CS4, participants were asked to assess the following treatment options according to their preferences:

1. Tooth retention using non-surgical endodontic retreatment followed by a coronal restoration;
2. Tooth retention using apicoectomy followed by a coronal restoration;
3. Tooth extraction followed by replacement with ISC;
4. Tooth extraction followed by replacement with FDP;
5. Tooth extraction followed by replacement with RDP;
6. Tooth extraction followed by no replacement.

All fictive patients presented in the case scenarios had private health insurance and no medical records or underlying diseases to eliminate bias in decision-making caused by financial or systemic health situations of the patients. The response scale for each treatment option displayed three choices: ‘very appropriate’, ‘appropriate’, and ‘not appropriate’.

### 2.2. Sample Size Calculation

To determine the number of responding dentists needed for a significant database, the following conditions were defined for the sample size calculation:

1. Number of sent emails to registered dentists in the northern state of Schleswig-Holstein, Germany (*N* = 2270);
2. A confidence level of 95%;
3. A margin of error of 6%.

Based on these conditions it was determined that within Schleswig-Holstein in northern Germany at least 239 dentists were needed for a statistically significant sample size.

### 2.3. Statistical Analysis

Data from the online questionnaire was digitally recorded by the web-based survey tool and analyzed using PASW (Predictive Analysis Software) Statistics for Windows software (SPSS Inc., Version 18.0.0, Chicago, Ill., USA). The Shapiro–Wilk-Test was performed to test for normality of the data. Data was not normally distributed. Statistical comparisons of the different ratings of treatment choices by the participants within each case scenario were performed using the non-parametric Friedman test. The mean rating value points of each treatment option was evaluated by giving value points (VP) to each answer of the participants (“very appropriate” = 1VP; “appropriate” = 2VP and “not appropriate” = 3VP). Chi-square tests for independence were used for association analysis between participant-related factors (gender, work experience, location of practice, treatment spectrum in the dental practice and percentage of privately insured patients) and the most preferred treatment options (smallest mean rating value points) stated in each case scenario of the survey. Statistical significance was set at *p* < 0.05.

## 3. Results

### 3.1. Participation and Sociodemographic Data 

A total of 366 dentists participated in the survey with 260 completed questionnaires resulting in a statistically significant sample. Participants included female and male dentists with different years of work experience. The majority of respondents (71.5%) were self-employed in their practice. Most of the participants were general practitioners with few specialized dentists mainly in the fields of implantology, endodontics, and periodontology.

The locations of the respondents’ workplaces were evenly distributed with the smallest group located in the suburban area (18.5%).

Slightly more than one third (34.6%) of the respondents stated a proportion of 20–40% of privately insured patients, while the majority of participants indicated to have a smaller percentage of statutory insured patients (Table 1)

Statements about the provided treatment services showed varying distributions of treatment spectra (TS1–TS4) performed in the dentists’ workplaces (Table 2).

### 3.2. Rating of the Case Scenarios

Tooth retention using RCT followed by a coronal restoration was the first-ranked treatment option in CS1 (Table 3) and CS2 (Table 4), while tooth retention using non-surgical endodontic retreatment followed by a coronal restoration was the first-ranked treatment option in CS3 (Table 5) and CS4 (Table 6). Second choice treatment preferences in all case scenarios were either tooth retention using apicoectomy followed by a coronal restoration or tooth extraction followed by replacement with ISC (Table 3, Table 4, Table 5 and Table 6)

Statistical analysis displayed statistically significant differences (*p* < 0.05) between all four case scenarios’ mean rating values of the different treatment options (Table 3, Table 4, Table 5 and Table 6).

For CS2, the factors’ analysis showed a significant positive correlation (*p* < 0.05) between the first-ranked treatment option (tooth retention after RCT/retreatment) and increasing work experience of the dentists. Significantly more female dentists (83%) also decided to preserve the tooth in CS2 by endodontic treatment, compared to the male counterpart (72.5%). For CS3 and CS4, a significant positive correlation was observed between the first-ranked treatment option (tooth retention by non-surgical endodontic retreatment) and higher numbers of privately insured patients at the dental practice (Table 7). 

## 4. Discussion

Recently, there has been rising attention describing the consequences of disease or medical decisions by measuring its effect on patients’ daily living and quality of life [5]. Numerous efforts have been employed to evaluate the relationship between quality of life and health related factors, leading to the widely spread term health-related quality of life. Evaluations of health-related quality of life have been widespread in medical areas as well as within fields of dentistry and oral health. Oral health is an essential part of general health, therefore it contributes extensively to health-related quality of life at biological, psychological, as well as social levels. Several studies previously reported the negative effect of tooth loss on the quality of life of dental patients [19]. Treatment of AP, a very prevalent dental condition, is a complex process in which dentists are regularly confronted with the difficult question of whether to save the affected teeth or to extract them [4,20]. It has been described as the first stage of all other developments of periapical endodontic lesions, either in an acute or chronic direction and its treatment modalities have been thoroughly discussed in various studies [21]. Nevertheless, up to the present time there is no definite treatment protocol or recommendation for teeth with AP. Thus, this survey aimed to evaluate for the first time German dentists’ preferences in northern Germany for the treatment of teeth with AP.

In this survey 2270 dentists from the state of Schleswig-Holstein in northern Germany were invited to participate via the dental regulatory body’s email register and a statistically significant sample size of 260 dentists completed the online questionnaire, displaying a similar response rate (11.4%) among the dental community reported in other studies in northern Germany [22,23]. Most of the non-responders did not contribute for no explicit reason or due to the absence of a reward, as observed in similar studies [4]. Sociodemographic data of the participants showed a similar gender distribution compared to the dental population in Germany and Europe (Table 1) [24,25]. The majority of the survey participants were dentists with no specialization (general practitioners), currently practicing in their own practice with a work experience of more than 20 years (Table 1). This corresponds to the reported average age and equivalent professional characteristics among dentists in Schleswig-Holstein, Germany [22,23]. Almost all participants of the study performed RCT in their workplace but only half of them performed implant treatments and one third of them performed endodontic microsurgeries (Table 1). This can be explained as RCT are commonly performed by general practitioners and are considered one of the basic dental treatments in Germany. Implantology and endodontic microsurgery, however, are more specialized treatment options that require further education, practical experience, and patients who are able to afford these procedures financially [26]. These results revealed that for most sociodemographic data that could be compared, variances between the relatively small study sample and its counterpart of dentists in northern Germany were rather non-significant, suggesting a minimal response bias. Consequently, the study participants can be considered as an acceptable and representative sample of Schleswig-Holstein’s dentists. 

Making treatment decisions is considered one of the most difficult aspects challenging any healthcare professional [27]. It shows a high level of complexity due to the expectations of the patient, and the contemplations the health care professional must make in selecting a treatment that is not only effective, but additionally maximizes benefits and health-related quality of life of the patient while minimizing risks. Often the construction of a decision selecting a definite treatment option may be linked to empirical principles or experiences of the dentist, which explains why dental education or training affects the treatment choices of oral healthcare professionals [28]. This effect may arise from differences in the diagnosis or identification of certain conditions [29], the preferred decisions to treat these conditions according to the dentist’s awareness of the risks and progression of the disease, as well as the most appropriate procedure applied for treatment [30]. Furthermore, the practice of dentistry imitates market principles in which the patients’ or clinicians’ sensitivity to financial burdens, costs, and health insurance coverage of different treatments besides the patients’ cooperation might influence the treatment decision by dentists and their patients [31,32]. Treatment recommendations or decisions might also be affected by the medical and dental history of the patient [33], as well as the specific tooth or surface to be treated [34], in which the dental community also tends to disagree. Indeed, all factors affecting the treatment decisions by oral healthcare professionals can be divided into patient-related (PRF), practice/education-related (ERF), and tooth-related factors (TRF), providing the base for dentists in their judgements and treatment planning [35,36]. In the current survey the practice/education- and tooth-related levels were addressed, while patient-related factors were excluded as explained above to eliminate predisposition in decision-making produced by financial or systemic health circumstances of the fictive patients.

From among the treatment options study participants could select, tooth retention using RCT or non-surgical endodontic retreatment followed by a coronal restoration was noticeably the first-ranked treatment option for all four case scenarios (Table 3, Table 4, Table 5 and Table 6). This expresses a strong tendency towards tooth preservation when considering the treatment options for AP among dentists in Schleswig-Holstein addressing the contemporary dilemma facing dentists worldwide, whether to treat the teeth endodontically or extract them for a prosthetic procedure. This result can be expected due to the priority given by the German oral health system in the last decade to preventive and conservative dental care instead of prosthetic treatment, supported by financial incentives for tooth preserving measures and introduction of preventive and conservative dentistry principles in the dental curricula and education (ERF) [37]. Similar results were described previously in Canada [10], as well as South Korea [4]. Nevertheless, these surveys additionally reported a correlation between treatment preferences and the dental specialization. Since in Germany, as observed in this survey, the number of dental specialists is considered lower than of North American and other international dental societies and there are fewer state-recognized, post-graduate specialization programs, a differentiation of this kind was not feasible in the current investigation [26,38]. Unlike the first-ranked treatment preference, the second-rated treatment option in all case scenarios showed varying answers of the participating dentists. However, these choices seem to be affected mainly by the tooth location and difficulty of the treatment (TRF). For anterior teeth (CS1 and CS3), the second-rated preference was tooth retention using apicoectomy followed by a coronal restoration, while for posterior teeth (CS2 and CS4), the treatment option was tooth extraction followed by replacement with ISC (Table 3, Table 4, Table 5 and Table 6). This finding concurs with previously published results and can be explained by the fact that apicoectomy on a posterior tooth is technically challenging (especially on the palatal root apex) and displays lower success rates than the anterior region due to access difficulties and complicated anatomical properties of the posterior teeth [10,39,40]. Further association analysis of the first-ranked treatment options revealed significant positive correlations between the treatments of choice in CS2 and CS4 (Table 7) and higher work experience. More years of work experience can be associated with advanced practical skills [41]. This could explain the enhanced preference for root canal (re)treatment on posterior teeth which often present a more challenging root canal system than anterior teeth [40]. Another observed significance correlated the preferred treatment in CS3 and CS4 positively (Table 7) to the higher percentage of privately insured patients in the dentist’s practice. Dental practices with a higher percentage of privately insured patients might have more specialized technical equipment as endodontic microscopes, as well as higher skills using them to perform root canal retreatment [42,43]. This may be attributed to the fact that privately insured patients find it easier to finance this complex and cost-intensive treatment option which is often not covered by statutory insurance. Both observed associations can correspondingly be ascribed to all three mentioned levels of factors affecting the treatment decisions in dental practice (PRF, ERF, and TRF). Female participants also presented a significantly higher tendency to preserve posterior teeth by RCT in CS2 only when compared to male dentists (Table 7). This corresponds to previous findings describing gender as one of the potential dentist-related associated factors connected with the decision to retain compromised teeth and showing a higher inclination of female clinicians towards tooth preservation [35,44]. Nevertheless, gender as an influencing factor on clinical treatment decisions seems to be less important than the other factors being only significant in one CS in the current study and similarly observed and explained in previous investigations [10,12].

As clinical dentistry is becoming progressively complex and patients more educated, evidence-based dental care is nowadays looked upon as the “gold standard” of dental treatment worldwide [30]. This entitles the patients to receive correct information about state-of-the-art treatment techniques and possibilities from their dentist, as clinician’s opinions can affect the final decision of their patient [45,46]. Although many participants of this study displayed an obvious tendency following evidence-based treatment of AP and tooth preservation, the results indicated some obsolete or non-evidence-based treatment recommendations. For example, almost half of the respondents in CS1 and CS2 (Table 3 and Table 4) have surprisingly ranked the treatment option “tooth retention using apicoectomy followed by a coronal restauration” as “(very) appropriate” although this option is not indicated scientifically until primary RCT has failed [47]. Such lack of consensus has already been reported for teeth with AP and previous RCT and endorses the described claims of subjective and inconsistent treatment recommendations [48,49]. Previously reported factors disturbing the adoption of modern and evidence-based dental technology and science in decision making, such as psychosocial and behavioral factors, as well as a complex interplay of perceived benefits might provide an explanation of this observation [50,51]. The outcomes of this survey reflect the current clinical situation of the dental society in Germany and worldwide, where, even though dentists are capable of saving a tooth with AP through endodontic treatment, some clinicians are more likely to extract the tooth for implantation or recommend other non-evidence-based treatment options. Despite academic and dental associations’ efforts to increase clinical agreement concerning the treatment of AP, a consensus has still not been reached and many dentists continue to depend only on their clinical experience and other personalized factors. Patients generally prefer to save their restorable teeth over tooth extraction [52]. Providing the best care and treatment to patients according to their preferences within the evidence-based scope of treatment is the chief duty of every clinician. Consequently, continuous and proper education and dental training should be offered to the dental society. This could be fulfilled by reviewing and re-evaluating the endodontic education programs on under- and postgraduate levels, as well as targeting the diagnostic and clinical skills of the dentists and also improving their self-efficacy and self-perceived competence [53]. Equally important, advanced diagnostic methods, such as artificial intelligence-guided techniques, should be introduced to support the clinical decision-making process based on scientific evidence and train the dentists to correctly interpret and diagnose the conditions [54]. In addition, further evaluation and consideration of the factors affecting the treatment decisions of AP (ERF, PRF, and TRF) should be performed to establish scientifically-based treatment guidelines for AP and guarantee a dental health system allowing the correct treatment of the patients. 

### Limitations of the Study

The following limitations have to be acknowledged. One main disadvantage of this cross-sectional study is that the data analyzed are restricted to the available information. On the other hand, misdiagnoses and misinterpretations in this type of study are also known to be equally distributed. In this investigation the observed treatment decisions of German dentists cannot be attributed completely to the analyzed factors and socioenvironmental data. Examining further co-variables and conditions such as additional clinical case scenarios (including different teeth as premolars) and further sociodemographic observations (including age, financial challenges, and place of initial training) could play a key role influencing the outcomes of the survey. Moreover, the present investigation examined a relatively small sample of dentists in the state of Schleswig-Holstein in northern Germany. The results obtained may not be representative for other German states or generalizable on all dentists in Germany. However, it could serve as an indicator of German dentists’ main preferences due to the equality of dental education and training in Germany as explained in previous studies [24]. Furthermore, it may be important to add radiographic diagnosis and patient symptoms to the clinical case scenarios as they could be crucial to make the correct treatment decisions [4]. Yet, because of considerable inter- and intra-observer variability and radiographic interpretation divergences as previously reported in comparable studies related to endodontic treatment [10,55], radiographic images were intentionally left out in the case scenarios to avoid similar complications. Another significant limitation of this investigation is the potential presence of social desirability bias when answering the questions of the survey. Respondents may tend to deliver responses based on textbook recommendations, which might not necessarily reflect their actual clinical performs. This bias might also continue in spite of the confidentiality promised to participating dentists. Furthermore, the lack of knowledge about time from endodontic treatment to the present could also be considered a limiting factor of this investigation. It is also not known whether the apical status has improved or not after RCT. However, these conditions are also unknown among comparable studies, and the results from the current investigation are largely harmonious with other studies in the literature.

## 5. Conclusions

This survey investigated for the first time preferences of dentists in the state of Schleswig-Holstein in northern Germany for the treatment of AP and the factors affecting their decisions. The majority of dentists preferred tooth retention using RCT or non-surgical endodontic retreatment followed by a coronal restoration, while the less preferred option was tooth extraction and replacement with an ISC or apicoectomy followed by a coronal restoration. The observed results reflected a significant positive association between retention of posterior teeth using RCT or non-surgical endodontic retreatment and the work experience of the dentists, as well as the preference for retention of previously root canal filled teeth and the higher percentage of privately insured patients in the dentist’s practice.

The results reveal a lack of consensus regarding the indication of different treatment options for AP among the dentists in Germany and indicates a personalized, non-evidence-based decision making by some clinicians. To ensure correct evidence-based patient guidance throughout the treatment process, an establishment of definite treatment protocols for AP besides continuous advanced endodontic education and clinical training for general practitioners are recommended. Furthermore, other external factors affecting the treatment decisions on financial and insurance-related levels should be considered and re-evaluated to prevent any interference with the guidelines.

## Figures and Tables

**Table 1:** Sociodemographic and professional data of the survey participants.

Sociodemographic and Professional Characteristics	Distribution (*n* = 260)
Sex	
Male	149 (57.3%)
Female	111 (42.7%)
Work experience	
0–9 years	70 (26.9%)
10–19 years	56 (21.5%)
>19 years	134 (51.5%)
Employment status ^1^	
Currently practicing in own practice	186 (71.5%)
Employed dentist in (panel/private) practice	36 (13.8%)
Employed dentist during assistantship in (panel/private) practice	17 (6.5%)
Director/head of a university clinic	0 (0%)
Senior dentist at a university clinic	2 (0.8%)
Assistant dentist at a university clinic	12 (4.6%)
Medical officer (military)	3 (1.2%)
Not practicing/retired	5 (1.9)
Dental specialization ^1^	
No specialization/GP	250 (96.2%)
Orthodontist	0 (0%)
Oral surgery	6 (2.3%)
Periodontology	2 (0.8%)
Public health	0 (0%)
Maxillofacial surgery	2 (0.8%)
Certified focus of treatment ^1^	
Geriatric dentistry	4 (1.5%)
Endodontology	29 (11.2%)
Functional analysis/therapy	4 (1.5%)
Implantology	39 (15.0%)
Aesthetic dentistry	4 (1.5%)
Pediatric dentistry	8 (3.1%)
Laser dentistry	0 (0%)
Periodontology	21 (8.1%)
Prosthodontics	10 (3.8%)
Restorative dentistry	4 (1.5%)
Other dental specialization or focus of treatment	8 (3.1%)
Location of practice	
Inner city/commercial subcenter	61 (23.5%)
Urban area	75 (28.8%)
Suburban area	48 (18.5%)
Rural area	76 (29.2%)
Provided treatment services ^1^	
Root canal treatment	255 (98.1%)
Tooth extraction	249 (95.8%)
Implantology (with/without restoration)	131 (50.4%)
Non-surgical endodontic retreatment	214 (82.3%)
Endodontic microsurgery (e.g., apicoectomy)	86 (33.1%)
FDP	251 (96.5%)
RDP	243 (93.5%)
Privately insured patients	
0–20%	150 (57.7%)
20–40%	90 (34.6%)
40–60%	17 (6.5%)
60–80%	3 (1.2%)
80–100%	0 (0%)

^1^ The sum of this group may be more than *n* = 260 (100%) because multiple answers were possible. GP: General practitioner; FDP: Fixed dental prostheses; RDP: Removable dental prostheses.

**Table 2:** Proportions of the four subgroups based on categories of treatment spectrum performed in the dental practice.

Subgroup	Distribution (*n* = 260)
TS1: practicing endodontics but not implants	129 (49.6%)
TS2: practicing implants but not endodontics	5 (1.9%)
TS3: practicing both	126 (48.5%)
TS4: practicing neither	0 (0%)

**Table 3:** Preferences of survey participants regarding case scenario 1 in %, mean rating value and results of the Friedman test comparing all treatment choices.

Case Scenario 1: AP on a Permanent, Anterior Tooth with no Root Canal Filling					
Treatment Choices	Very appropriate (1 value point)	Appropriate (2 value points)	Not appropriate (3 value points)	Mean rating value	Friedman test
Tooth retention using RCT followed by a coronal restoration	84.6	15.0	0.4	1.2	*p* < 0.001
Tooth retention using apicoectomy followed by a coronal restoration	9.2	42.7	48.1	2.4	
Tooth extraction followed by replacement with ISC	6.2	50.4	43.5	2.4	
Tooth extraction followed by replacement with FDP	1.9	36.9	61.2	2.6	
Tooth extraction followed by replacement with RDP	0.0	7.3	92.7	2.9	
Tooth extraction followed by no replacement	0.0	2.3	97.7	3.0	

AP: Apical periodontitis. RCT: Root canal treatment. ISC: Implant supported crown. FDP: Fixed dental prostheses. RDP: Removable dental prostheses.

**Table 4:** Pre-preferences of survey participants regarding case scenario 2 in %, mean rating value and results of the Friedman test comparing all treatment choices.

Case Scenario 2: AP on a Permanent, Posterior Tooth with no Root Canal Filling					
Treatment Choices	Very appropriate (1 value point)	Appropriate (2 value points)	Not appropriate (3 value points)	Mean rating value	Friedman test
Tooth retention using RCT followed by a coronal restoration	77.3	21.5	1.2	1.2	*p* < 0.001
Tooth extraction followed by replacement with ISC	12.7	54.2	33.1	2.2	
Tooth retention using apicoectomy followed by a coronal restoration	6.9	44.6	48.5	2.4	
Tooth extraction followed by replacement with FDP	4.6	44.2	51.2	2.5	
Tooth extraction followed by no replacement	0.4	26.2	73.5	2.7	
Tooth extraction followed by replacement with RDP	0.4	16.2	83.5	2.8	

**Table 5:** Preferences of survey participants regarding case scenario 3 in %, mean rating value and results of the Friedman test comparing all treatment choices.

Case Scenario 3: AP on a Permanent, Anterior Tooth with Root Canal Filling					
Treatment Choices	very appropriate (1 value point)	Appropriate (2 value points)	not appropriate (3 value points)	MEAN rating value	Friedman test
Tooth retention using non-surgical endodontic retreatment followed by a coronal restoration	76.5	23.1	0.4	1.2	*p* < 0.001
Tooth retention using apicoectomy followed by a coronal restoration	16.9	43.1	40.0	2.2	
Tooth extraction followed by replacement with ISC	7.7	56.2	36.2	2.3	
Tooth extraction followed by replacement with FDP	2.3	42.7	55.0	2.5	
Tooth extraction followed by replacement with RDP	0.0	13.1	86.9	2.9	
Tooth extraction followed by no replacement	0.0	3.1	96.9	3.0	

**Table 6:** Preferences of survey participants regarding case scenario 4 in %, mean rating value and results of the Friedman test comparing all treatment choices.

Case Scenario 4: AP on a Permanent, Posterior Tooth with Root Canal Filling					
Treatment Choices	Very appropriate (1 value point)	Appropriate (2 value points)	Not appropriate (3 value points)	Mean rating value	Friedman test
Tooth retention using non-surgical endodontic retreatment followed by a coronal restoration	63.5	35.4	1.2	1.4	*p* < 0.001
Tooth extraction followed by replacement with ISC	14.2	56.5	29.2	2.2	
Tooth retention using apicoectomy followed by a coronal restoration	12.7	45.4	41.9	2.3	
Tooth extraction followed by replacement with FDP	4.6	43.5	51.9	2.5	
Tooth extraction followed by no replacement	0.8	26.5	72.7	2.7	
Tooth extraction followed by replacement with RDP	0.4	17.7	81.9	2.8	

**Table 7:** *p*-value of statistical association analysis by Chi-squared tests between the first-ranked treatment option of the four different case scenarios and different factors.

Case Scenarios	Gender	Work Experience	Location of the Practice	Category of Performed Treatment Spectrum	Percentage of Privately Insured Patients
Case scenario 1	0.29	0.11	0.32	0.52	0.42
Case scenario 2	0.05	0.01	0.94	0.80	0.92
Case scenario 3	0.09	0.07	0.34	0.15	<0.001
Case scenario 4	0.23	0.04	0.62	0.52	<0.001

The background color marks the significance.
